# A Novel Single-Dose Dengue Subunit Vaccine Induces Memory Immune Responses

**DOI:** 10.1371/journal.pone.0023319

**Published:** 2011-08-03

**Authors:** Chen-Yi Chiang, Shih-Jen Liu, Jy-Ping Tsai, Yi-Shiuan Li, Mei-Yu Chen, Hsueh-Hung Liu, Pele Chong, Chih-Hsiang Leng, Hsin-Wei Chen

**Affiliations:** 1 Vaccine Research and Development Center, National Health Research Institutes, Zhunan, Miaoli, Taiwan; 2 Graduate Institute of Immunology, China Medical University, Taichung, Taiwan; Agency for Science, Technology and Research - Singapore Immunology Network, Singapore

## Abstract

To protect against dengue viral infection, a novel lipidated dengue subunit vaccine was rationally designed to contain the consensus amino acid sequences derived from four serotypes of dengue viruses. We found that the lipidated consensus dengue virus envelope protein domain III (LcED III) is capable of activating antigen-presenting cells and enhancing cellular and humoral immune responses. A single-dose of LcED III immunization in mice without extra adjuvant formulation is sufficient to elicit neutralizing antibodies against all four serotypes of dengue viruses. In addition, strong memory responses were elicited in mice immunized with a single-dose of LcED III. Quick, anamnestic neutralizing antibody responses to a live dengue virus challenge were elicited at week 28 post-immunization. These results demonstrate the promising possibility of a future successful tetravalent vaccine against dengue viral infections that utilizes one-dose vaccination with LcED III.

## Introduction

The dengue viruses (DV) are members of the Flavivirus genus of the Flaviviridae family. There are four antigenically different serotypes (DV-1 through DV-4) of DV [1]. Each of the four serotypes of dengue viral infection is able to cause dengue fever, which is generally a self-limited febrile illness. However, certain dengue-infected individuals develop the life-threatening dengue hemorrhagic fever (DHF) or dengue shock syndrome (DSS) [2], [3]. The pathogeneses of DHF and DSS are complicated and still not fully understood. It is generally accepted that an effective dengue vaccine must provide concrete and long-lasting cross-protection against all four serotypes of DV.

In the past several decades, enormous efforts have been put into the development of dengue vaccines to combat the disease [4], [5]. These efforts include vaccines utilizing live attenuated strains of all four dengue viral serotypes [6]–[9], inactivated whole virions [10], [11], chimeric or genetically engineered strains [12]–[15], virally vectors [16]–[18], naked DNA [19]–[21], and recombinant subunits [10], [22]–[26]. Although some level of success has been attained, several obvious obstacles still exist. For example, live attenuated dengue vaccines must elicit appropriately balanced tetravalent immunity, and they must be characterized by a good safety profile. The inactivated whole virion dengue vaccines were restricted by the low virus yields in typical dengue viral cultures. Presently, no licensed dengue vaccine is available.

Recombinant subunit dengue vaccines may provide significant benefits over other approaches. For instance, subunit vaccines eliminate exposure to the virus, which means they are safer, and they are more easily manipulated through dose adjustments to obtain a balanced immune response to all four serotypes of DV. However, subunit vaccines are recognized as poor immunogens, they require several immunizations and they must be formulated with a strong adjuvant to generate a sufficient immune response [10], [22]–[29].

The crystal structure of the envelope protein from DV revealed that it contains three distinct domains [30]–[32]. Domain III of the dengue envelope protein has been associated with receptor binding [33], [34], and it contains several neutralizing epitopes [35]–[40]. These results suggest that domain III of the dengue virus envelope protein is a promising subunit vaccine candidate. In our previous study [25], we described the development of a novel subunit dengue vaccine candidate comprising a consensus dengue virus envelope protein domain III (cED III). Mice immunized with recombinant cED III formulated with aluminum phosphate were able to elicit neutralizing antibodies against the four serotypes of dengue virus.

To avoid the inherent problems of subunit dengue vaccines (poor immunogenicity, requirement of adjuvant, and multiple immunizations), we established a novel platform technology that can express high levels of recombinant lipoproteins with intrinsic adjuvant properties [41]. In this study, we demonstrated that lipidated cED III (LcED III) was able to induce neutralizing antibodies against all serotypes of DV without the use of exogenous adjuvant. Moreover, a single dose of LcED III was capable of inducing strong neutralizing antibody memory responses.

## Results

### LcED III activates macrophages and up-regulate CD40, MHC II, and costiumlatory molecules expression

**Figure 1:**
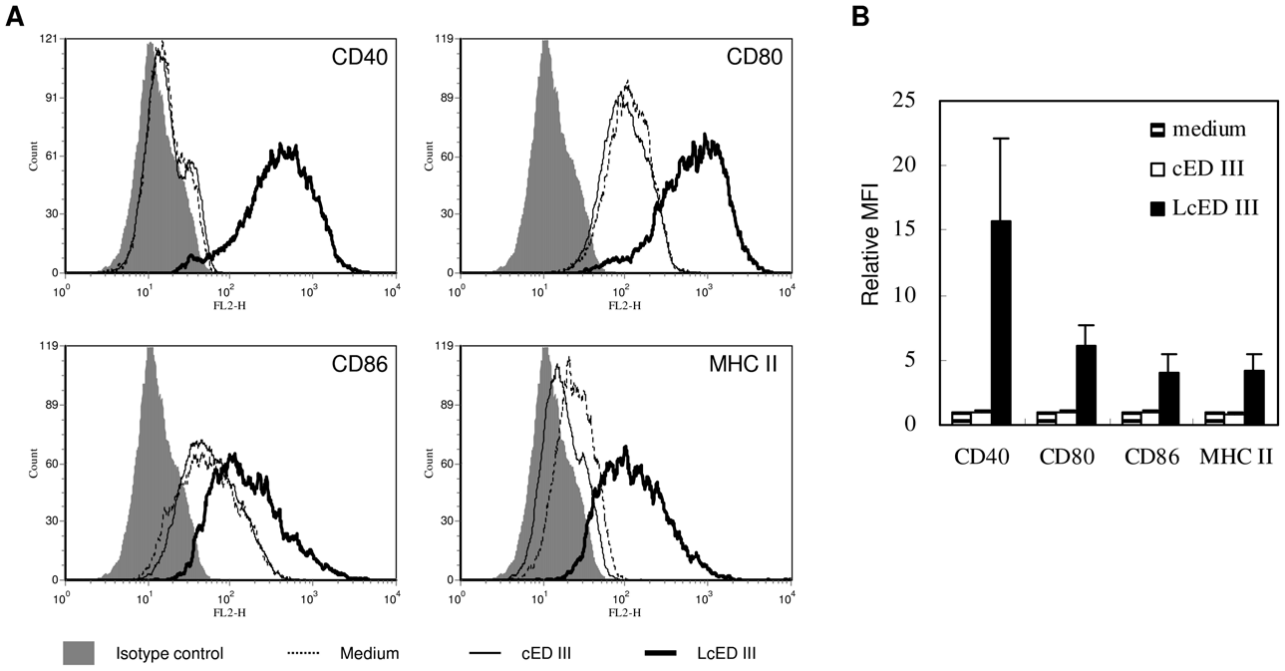
Effects of LcED III on the activation of RAW 264.7 macrophage cells. (A) RAW 264.7 macrophage cells were cultured either in media alone or in media supplemented with cED III or LcED III at 10 µg/mL. After incubation for 16 hours, the surface markers CD40, CD80, CD86, and MHC II were analyzed using flow cytometry. A representative experiment is shown. (B) The mean fluorescence intensity (MFI) for cells cultured in media alone was defined as the basal expression level. Relative MFIs were plotted. The means and standard deviations (SD) from three independent experiments are shown.

Lipoproteins or lipopeptides have been shown to activate antigen-presenting cells by triggering toll-like receptors [42]–[45]. To analyze the functional activity of LcED III, RAW 264.7 macrophage cells were stimulated with cED III or LcED III at 10 µg/mL for 16 hours. The expression levels of CD40, CD80, CD86, or MHC II on RAW 264.7 macrophage cells were analyzed by flow cytometry. As shown in Fig. 1A, LcED III up-regulated the expression of CD40, CD80, CD86, and MHC II, while cED III was ineffective at up-regulating these molecules. The mean fluorescence intensities (MFIs) obtained from cells cultured in medium alone were used to determine basal expression levels and were defined as 1. The relative MFIs from three independent experiments are summarized in Fig. 1B. LcED III stimulated macrophages to up-regulate CD40, CD80, CD86, and MHC II expression at levels 4- to 16-fold higher compared to those stimulated by cED III or medium alone.

### LcED III enhances T- and B-cell immune responses

**Figure 2:**
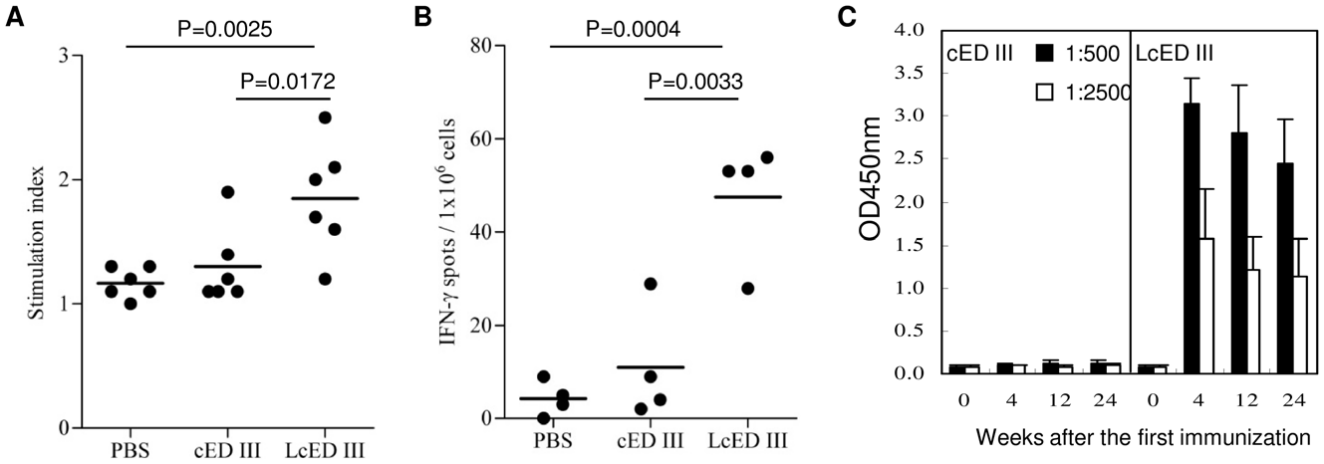
LcED III augments cellular and humoral immune responses. BALB/c mice were immunized subcutaneously with 20 µg of cED III or LcED III two times at a two-week interval. (A) Seven days after the last immunization, splenocytes were incubated with or without cED III (10 µg/mL). The stimulation index (SI) is defined as the ratio of the mean counts per minute (cpm) with cED III stimulation to the mean cpm without antigen stimulation. Results were pooled from two independent experiments. P values were calculated using an unpaired one-tailed Student's *t*-test. (B) The frequencies of IFN-γ-secreting cells in spleens were evaluated by mouse IFN-γ ELISPOT kits. Results are representative of two independent experiments. P values were calculated using the unpaired one-tailed Student's *t*-test. (C) Sera were collected at the indicated time points after the first immunization. Serum samples were diluted 1∶500 and 1∶2500 in PBS. Antibody responses against cED III were evaluated by ELISA. Pre-immune sera (week 0) were collected and used as basal levels. Results are plotted as the mean of optical density values plus standard deviation (n = 5).

Because recombinant LcED III up-regulated the expression of CD40, MHC II, and costimulatory molecules on macrophages *in vitro*, we evaluated the immune responses elicited by LcED III. Groups of BALB/c mice were immunized with cED III or LcED III two times at a two-week interval. Animals immunized with PBS alone served as negative controls. One week after the last immunization, splenocytes were examined for proliferative capacity and secretion of IFN-γ in response to cED III stimulation for 4 days. Fig. 2A shows that mice immunized with cED III did not develop a notable antigen-specific proliferative response. The stimulation index (SI) of the cells from these mice was comparable to the SI obtained from the splenocytes derived from mice immunized with PBS. In contrast, significant T-cell proliferation was elicited in mice immunized with LcED III. Moreover, the splenocytes obtained from LcED III-immunized mice contained a substantial number of IFN-γ-producing spots, which were significantly more numerous than in the splencoytes obtained from cED III- or PBS-immunized mice (Fig. 2B).

Next, we examined the IgG antibody responses following two doses (20 µg per dose) of cED III or LcED III vaccination. Serum samples were collected from the immunized mice at different time points, as indicated in Fig. 2C. Negligible levels of anti-cED III IgG antibodies were detected in mice immunized with cED III alone. However, mice immunized with LcED III were able to generate anti-cED III IgG antibodies. Importantly, substantial levels of anti-cED III IgG antibodies were maintained for over 24 weeks after the priming.

**Figure 3:**
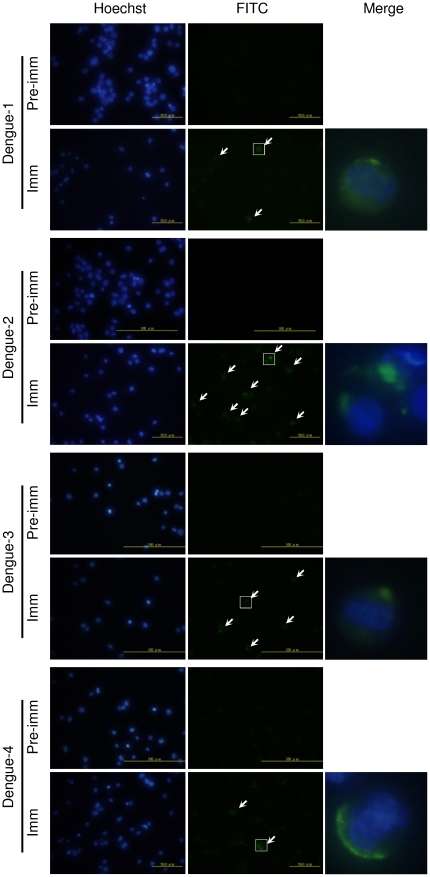
Antibodies induced by immunization with LcED III recognize all four dengue viruses. K562 cells were infected with each of the four dengue viruses as indicated. Three days after infection, the virus-infected cells were fixed and probed with either sera drawn before (Pre-imm) or after (Imm) immunizaiton with LcED III. Cellular DNA was visualized by Hoechst stains. The virus-infected cells were indicated by arrows. The right panels show the close-up images of merging Hoechst and FITC stains of the marked areas.

As the preceding experiments show that the LcED III can induce stronger antibody responses than cED III. The question to be raised at this point, will these antibodies recognize the four serotypes of dengue viruses? To address this, we performed indirect immunofluorescence staining to analyze the antibodies in sera of mice immunized with LcED III. As shown in Fig. 3, pre-immune serum did not produce immunofluorescence reactivity with any of the four dengue viruses. However, immune sera recognized all four dengue viruses as evidenced by immunofluorescence spectra (Fig. 3). These results suggest that antibodies induced by vaccination with LcED III can react with all four dengue viruses.

### LcED III elicits cross-neutralizing antibodies and memory immunity

**Figure 4:**
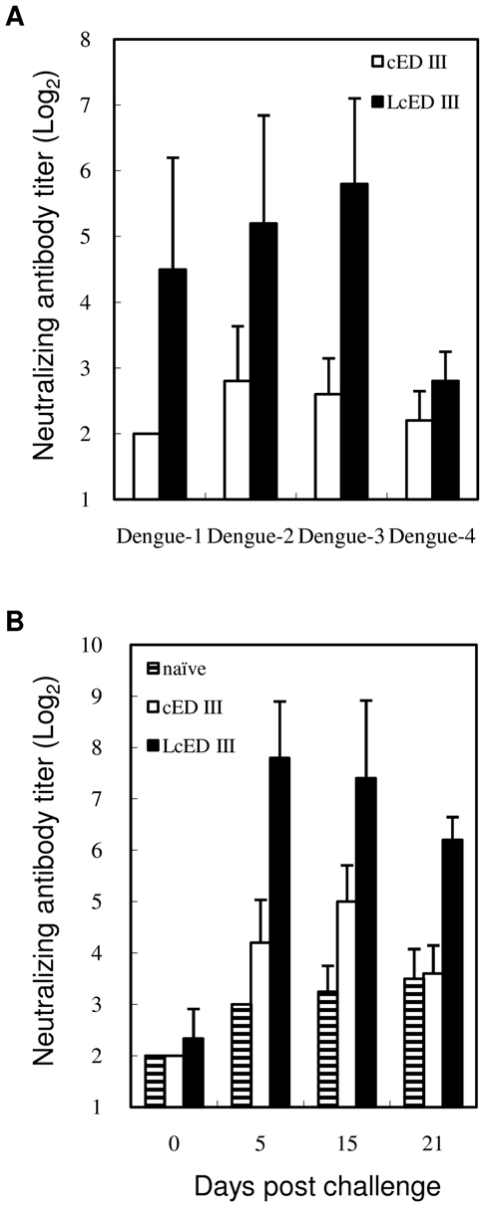
Induction of cross-neutralizing antibodies and memory neutralizing antibodies by vaccination with LcED III. BALB/c mice (n = 5) were immunized subcutaneously with 20 µg of cED III or LcED III two times at a two-week interval. (A) Sera samples were collected two weeks after the last immunization. (B) Immunized mice were inoculated intraperitoneally with 5×10^6^ FFU of live dengue-2 virus at week 28 post-priming. Sera obtained from naïve mice inoculated with live dengue-2 virus served as controls. Serum samples were collected from each mouse to evaluate neutralization of the infectivity of dengue virus by FRNT. The neutralizing antibody titer was calculated as the reciprocal of the highest dilution that resulted in a 40% reduction of FFU compared to that of a control that consisted of treatment with virus and pre-immunization sera. Values are representative of two independent experiments.

The major objective of this study was to explore whether LcED III could induce cross-neutralizing antibody responses. To analyze the neutralizing ability against the 4 serotypes of DV induced by vaccination, the antisera from each mouse immunized with cED III or LcED III were collected and determined the neutralizing antibody titers by focus reduction neutralization tests (FRNT). As shown in Fig. 4A, neutralizing antibody activities were not detected in mice immunized with cED III. Remarkably, antisera obtained from LcED III immunized mice were capable of blocking all 4 serotypes of dengue viral infection *in vitro*. The geometric mean neutralizing antibody titers against dengue-1, dengue-2, dengue-3, and dengue-4 were 2^4.5^, 2^5.2^, 2^5.8^, and 2^2.8^, respectively. These results indicate that LcED III alone is able to induce neutralizing antibodies that can concurrently inhibit dengue-1, dengue-2, dengue-3, and dengue-4 viral infection.

To test the durability of neutralizing antibody responses, mice were injected intraperitoneally with 5×10^6^ focus-forming units (FFU) of live dengue-2 virus at week 28 after the first immunization. The sera from mice immunized with cED III and challenged with dengue-2 induced 2^4.2^, 2^5.0^, and 2^3.6^ neutralizing antibody titers at 5, 15, and 21 days post-viral challenge, respectively. These neutralizing capacities were equivalent to the sera from naïve mice challenged with live DV. Notably, we observed over a 16-fold increase in the levels of neutralizing antibodies against dengue-2 at 5 days post-viral challenge. The neutralizing antibody titers were found to be >2^6^ at all time points (5, 15, and 21 days post-viral challenge) examined (Fig. 4B). These results provide tangible evidence that an efficient anamnestic neutralizing antibody response is induced in mice vaccinated with LcED III.

### A single dose of LcED III is able to induce anamnestic neutralizing antibody responses

**Figure 5:**
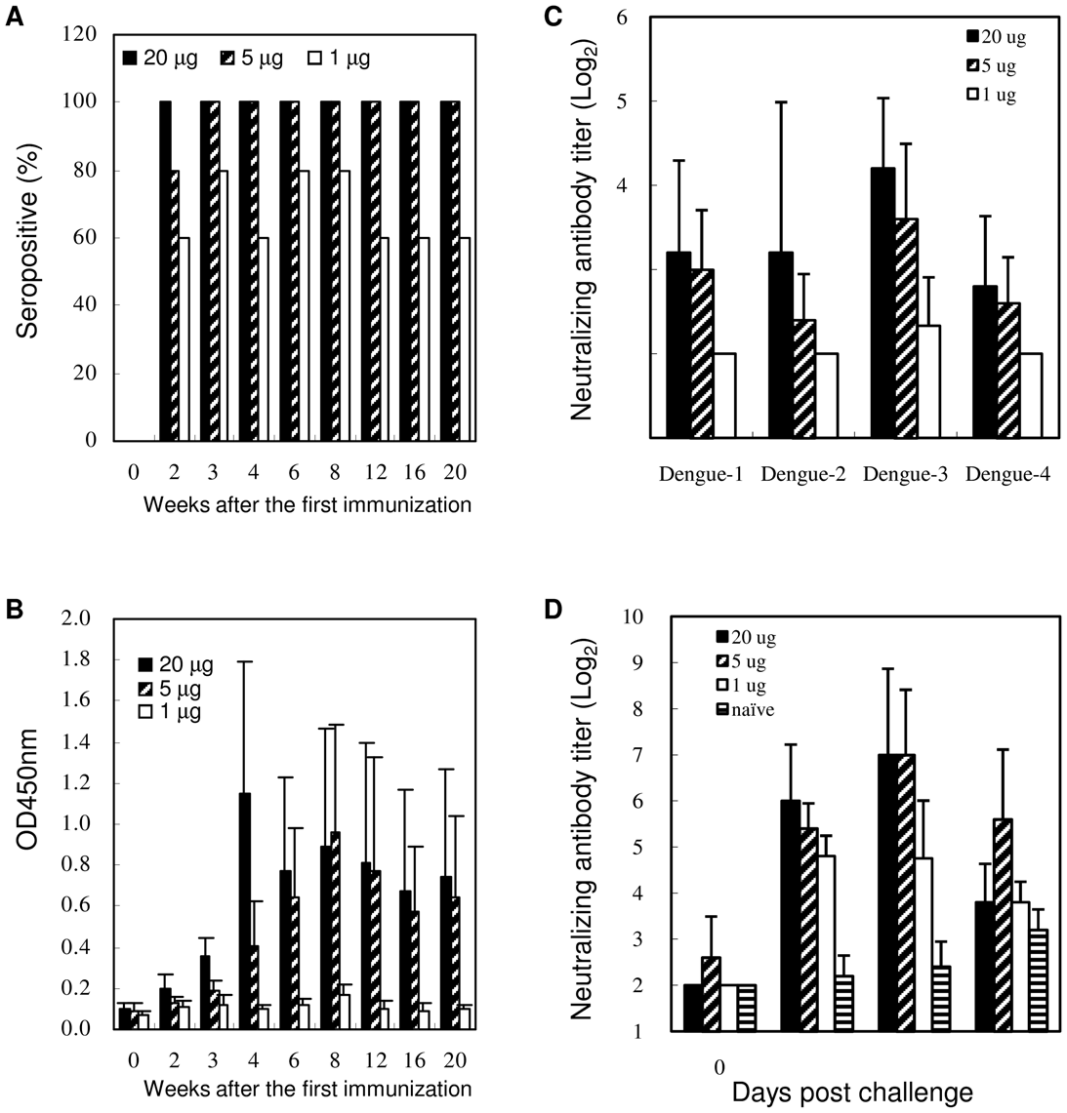
A single-dose of LcED III is able to induce cross-neutralizing antibodies and memory neutralizing antibodies. BALB/c mice (n = 5) were immunized once subcutaneously with 1, 5, or 20 µg of LcED III. (A) Seroconversion rates and (B) the optical density of serum samples at a 2500-fold dilution versus time after immunization are plotted. (C) Sera samples were collected four weeks after the immunization. The neutralizing antibody titer of the sera in each mouse was determined by FRNT. (D) Immunized mice were challenged intraperitoneally with 5×10^6^ FFU of live dengue-2 virus at week 28 post-priming. Sera obtained from naïve mice inoculated with live dengue-2 virus served as controls. Serum samples were collected in each mouse to evaluate neutralization of the infectivity of dengue virus by FRNT. The neutralizing antibody titer was calculated as the reciprocal of the highest dilution that resulted in a 40% reduction of FFU compared to that of a control that consisted of treatment with virus and pre-immunization sera.

To evaluate the potency of LcED III, groups of BALB/c mice were immunized with a single-dose vaccine containing various amounts (20, 5, or 1 µg) of LcED III. The sera from immunized mice were analyzed for the presence of cED III-specific antibodies or neutralizing capacity. The time course of seroconversion rates are shown in Fig. 5A. A seropositive sample is defined as when the optical density of the sample at a 100-fold dilution is more than two times higher than that of pre-immune serum. Only 60–80% of the mice vaccinated with 1 µg of LcED III were seropositive. Interestingly, the mice that received either 5 or 20 µg of LcED III generated high levels of anti-cED III IgG antibodies. All animals were seroconverted, and their antibodies endured over 20 weeks. Fig. 5B shows the time course of optical density obtained from the reactivity of serum samples with cED III at a 2500-fold dilution after the immunization. In summary, the mice that received 1 µg of LcED III generated low levels of anti-cED III IgG antibodies. Significant antibody responses were generated in mice immunized with either 20 or 5 µg of LcED III.

The serum samples were collected at week 4, and the neutralizing capacity against the 4 serotypes of dengue virus was examined. As shown in Fig. 5C, no significant levels of neutralizing antibodies were detected in the sera obtained from mice immunized with 1 µg of LcED III. Remarkably, mice immunized with a single dose of 20 µg of LcED III were able to generate cross-neutralizing antibodies against the 4 serotypes of DV.

To evaluate the memory neutralizing antibody responses, mice were injected intraperitoneally with 5×10^6^ FFU of live dengue-2 virus at week 28 after the immunization. No neutralizing antibody activity was detected in the sera obtained from naïve mice until 19 days after viral challenge (Fig. 5D). In contrast, geometric mean neutralizing antibody titers were detected and maintained at 2^3.8^–2^4.8^ in mice immunized with 1 µg of LcED III at all times that we examined. Moreover, mice immunized with 20 and 5 µg of LcED III were found to have quicker and stronger neutralizing antibody responses. The geometric mean neutralizing antibody titers of these two groups were determined to be 2^6^ and 2^5.4^ on the 4th day post-viral challenge, respectively. The geometric mean neutralizing antibody titers in the sera obtained from mice immunized with 20 and 5 µg LcED III both further increased to 2^7^ on the 12th day after viral challenge. These results indicate that a single dose of LcED III is able to induce a memory neutralizing antibody response.

## Discussion

The dengue viral envelope protein is responsible for viral attachment by binding to the cellular receptor. Domain III of the envelope protein was found to be involved in receptor binding [33], [34]. It has been demonstrated that envelope protein domain III is a leading target in dengue subunit vaccine development [46]. In our previous study [25], we designed a consensus amino acid sequence of domain III from the dengue envelope protein, and we used it successfully for dengue subunit vaccine development. Unfortunately, the cED III vaccine contained a problem inherent to subunit vaccines, poor immunogenicity. Without the addition of proper adjuvant, cED III alone induced low neutralizing antibody responses. To overcome this inherent problem of subunit vaccines, we developed LcED III as a novel dengue vaccine candidate that has a built-in intrinsic adjuvant. In this study, we demonstrated that LcED III stimulated macrophages and up-regulated the expression of CD40, MHC II, and costimulatory molecules *in vitro* (Fig. 1). These results are consistent with our previous findings in the bone marrow-derived dendritic cells [41], [42]. We then evaluated the capacity of LcED III to elicit cellular and humoral immune responses. As expected, LcED III is superior to cED III in its ability to induce cED III-specific T-cell proliferative responses, IFN-γ secretion, and IgG production (Fig. 2). Taken together, the results demonstrate that LcED III is able to activate antigen-presenting cells and enhance cellular and humoral immune responses.

After verifying that LcED III stimulates superior immune responses, we initiated experiments to study the capability of LcED III of inducing neutralizing antibodies against the 4 serotypes DV. LcED III alone, without exogenous adjuvant formulation, was able to induce cross-neutralizing antibody responses (Fig. 4A). Moreover, the neutralizing antibodies were evoked rapidly when the immunized mice were challenged with live DV (Fig. 4B). These results suggest that memory neutralizing antibody responses were elicited in mice immunized with LcED III.

It has been demonstrated that envelope protein domain III is a suitable candidate for a dengue subunit vaccine [46]. However, several vaccination doses and a proper adjuvant are required to obtain an effective immunity [22], [25]–[29]. The multiple doses usually result in an increase in vaccination cost, thus, a single-dose vaccine is more cost-effective than a multiple-dose vaccine. Our vaccine candidate, LcED III, is highly immunogenic (Fig. 5A and 5B). We demonstrated that a single dose, particularly without exogenous adjuvant formulation, is sufficient to stimulate neutralizing antibody responses (Fig. 5C). Importantly, the neutralizing antibodies were durable, and they could be recalled quickly. As shown in Fig. 5D, neutralizing antibodies were detected 4 days after viral challenge in the groups of mice immunized with a single dose of 1, 5, and 20 µg of LcED III, but they were not detectable in naïve mice challenged with the same titer of DV.

Although low cross-neutralizing antibody titers were induced by LcED III (Fig. 4A and 5C), substantial anamnestic neutralizing antibody responses were obtained in LcED III-immunized mice (Fig. 4B and 5D). In summary, we evaluated the novel LcED III as a potential dengue vaccine candidate. Overall, our results provide a new window of opportunity for dengue subunit vaccines, particularly for those based on dengue envelope protein domain III.

## Materials and Methods

### Ethics statement

All animal studies were approved by the Animal Committee of the National Health Research Institutes (Protocol No: NHRI-IACUC-095001) and were performed according to their guidelines.

### Expression and purification of recombinant cED III and LcED III

Preparation of cED III and LcED III were performed as previously described [25], [41]. Briefly, the cED III gene, either alone or fused with a lipidation signal DNA sequence, was cloned into the pET-22b(+) vector and expressed in *E. coli* BL21 (DE3) or C43 (DE3), respectively. Recombinant cED III and LcED III were purified by immobilized metal affinity chromatography, and the amount of residual LPS was negligible (<3 EU/mg) in both preparations.

### Virus

Four serotypes of the DV, namely, dengue-1/Hawaii, dengue-2/PL046, dengue-3/H-087, and dengue-4/H241, were used for this study. Virus propagation was performed in C6/36 cells, and virus titers were determined by focus-forming assays with BHK-21 cells.

### Mouse experiments

Five BALB/c mice (6–8 weeks of age) were immunized subcutaneously with cED III or LcED III (1–20 µg per dose). Mice were given one or two immunizations at a two-week interval with the same regimen. To detect the anamnestic response generated by immunization, immunized mice were inoculated intraperitoneally with 5×10^6^ FFU of live dengue-2 virus. Blood was collected from each mouse at different time points, as indicated. Sera were prepared and stored at −80°C until use.

### Lymphocyte proliferation assays

To determine whether cED III-specific lymphoproliferative responses were induced in immunized animals, spleens were removed one week after the last immunization to make single-cell suspensions. Splenocytes were seeded at a concentration of 2×10^5^ cells / well in 96-well plates and stimulated with cED III (10 µg/mL) for 4 days at 37°C in a 5% CO_2_ humidified incubator. During the final 18 hours of culture, 1 µCi of [^3^H]-thymidine was added to each well, and the cells were harvested using a FilterMate automatic cell harvester (Packard). The incorporated radioactivity was determined with a TopCount microplate scintillation counter (Packard). Con A (5 µg/mL) was included in the assay as a positive control. The induced stimulation index is defined as the ratio of the mean counts per minute (cpm) with cED III stimulation to the mean cpm without cED III stimulation.

### ELISPOT assays

The number of IFN-γ-producing cells was determined by mouse IFN-γ ELISPOT kits (eBioscience). All the assays were performed according to the procedures that were specified in the instructions included in the kits. Briefly, 96-well plates with PVDF membranes (Millipore) were coated with capture antibody and incubated at 4°C for 18 hours. The plates were washed twice and blocked with RPMI medium supplemented with fetal bovine serum (10%) for one hour to prevent nonspecific binding in later steps. Splenocytes were seeded at a concentration of 5×10^5^ cells / well and stimulated with cED III (10 µg/mL) for 4 days at 37°C in a 5% CO_2_ humidified incubator. After incubation, the cells were removed from the plates by washing three times with 0.05% (w/v) Tween 20 in PBS. A 100 µL aliquot of biotinylated detection antibody was added to each well. The plates were incubated at 37°C for 2 hours. The washing steps were repeated as above, and after a 45-minute incubation at room temperature with the avidin-horseradish peroxidase complex reagent, the plates were washed three times with 0.05% (w/v) Tween 20 in PBS and then three times with PBS alone. A 100 µL aliquot of 3-amine-9-ethyl carbazole (Sigma-Aldrich) staining solution was added to each well to develop the spots. The reaction was stopped after one hour by placing the plates under tap water. The spots were counted using an ELISPOT reader (Cellular Technology Ltd.).

### Measurement of antibody responses

The levels of anti-cED III IgG in the serum samples was determined by titrating the samples as previously described [25]. Briefly, purified cED III was coated on 96-well plates. Bound IgG was detected with horseradish peroxidase-conjugated goat anti-mouse IgG Fc. After the addition of 3, 3′, 5, 5′-tetramethylbenzidine (TMB), the absorbance was measured with an ELISA reader at 450 nm.

### Immunofluorescence assay

K562 cells were infected respectively with each of the four dengue virus serotypes. Three days after infection, viruses in the infected cells were detected by an indirect immunofluorescence assay, using mouse pre-immune and immune sera (from LcED III-immunized mice). Cellular DNA was labeled by Hoechst stains.

### Focus reduction neutralization tests (FRNT)

Antibody-mediated DV neutralization in BHK-21 cells was determined by FRNT as previously described [25]. Briefly, a monolayer of BHK-21 cells in 24-well plates was inoculated with DV that had been pre-mixed with pre-immunization or post-immunization sera to a final volume of 0.5 mL. The virus titer prior to pre-mixing was about 50 FFU per well. The pre-mixing was performed overnight at 4°C. Viral adsorption was allowed to proceed for 3 hours at 37°C. An overlay medium containing 2.5% fetal bovine serum and 0.8% methylcellulose in DMEM was added at the conclusion of adsorption. The infected monolayer was incubated at 37°C. After 72 to 120 hours of infection, the overlay medium was removed from the wells, and the BHK cells were washed with cold PBS. The cells were fixed for 15 min in 3.7% formaldehyde/PBS. After washing with PBS, the cells were permeabilized with 0.1% Nonidet P40/PBS for 15 min and blocked with 3% bovine serum albumin/PBS for 30 min. Infected cells were detected by a monoclonal anti-dengue antibody (American Type Culture Collection, No. HB-114). The monoclonal anti-dengue antibody reacted with all serotypes of the dengue virus. After washing with PBS, antibody-labeled cells were detected using a secondary antibody conjugated to horseradish peroxidase. The labeling was visualized using TMB. The FFU were counted, and the neutralizing antibody titer was calculated as the reciprocal of the highest dilution that produced a 40% reduction of FFU compared to control samples containing the virus and pre-immunization sera. The neutralizing antibody titer was designated as 2^2^ when neutralizing antibody titer was less than 2^3^.

### Statistical analyses

Statistical analyses were carried using GraphPad Prism version 5.02 (GraphPad Software, Inc.). Statistical significance of differences between groups was assessed using a one-tailed Student's *t*-test. Differences with a P value of less than 0.05 were considered statistically significant.
